# High biosorption of cationic dye onto a novel material based on paper mill sludge

**DOI:** 10.1038/s41598-023-43032-x

**Published:** 2023-09-23

**Authors:** Meriem Merah, Chahra Boudoukha, Antonio Avalos Ramirez, Mohamed Fahim Haroun, Samira Maane

**Affiliations:** 1 Department of Chemistry, Faculty of Sciences, University of Ferhat ABBAS Setif 1, 19000 El Bez, Algeria; 2 Department of Biochemistry, Faculty of Life Sciences, University of Ferhat ABBAS Setif 1, 19000 El Bez, Algeria; 3 Centre National en Électrochimie et en Technologies Environnementales, 2263 Avenue du College, Shawinigan, QC G9N 6V8 Canada; 4 Département de Génie Chimique et Génie Biotechnologique, Faculté de Génie, Université de Sherbrooke, 2500, Boul. de l’Université, Sherbrooke, QC J1K 2R1 Canada; 5 Laboratoire de Physique Quantique et Systèmes Dynamiques (LPQSD), University of Ferhat ABBAS Setif 1, 19000 El Bez Setif, Algeria

**Keywords:** Pollution remediation, Materials science, Sustainability

## Abstract

The valorization of paper mill sludge (PMS) is the main goal of this study. The emissions of PMS continue to increase at global scale, especially from packaging paper and board sectors. The raw sludge was used to prepare an adsorbent to remove toxic pollutants from wastewater, the methylene blue (MB), an organic dye. Firstly, the physico-chemical characterization of PMS was done determining the crystalline phases of PMS fibers, the content of main elements, and the pH zero point charge, which was determined at around pH 7. The adsorption of MB on PMS powder was studied at 18 °C with an agitation of 200 rpm, being the best operating conditions 30 min of contact time, 250 mg L^−1^ of initial MB concentration and 0.05 g in 25 mL of adsorbent dose. Experimental data of MB adsorption was fitted to Langmuir and Freundlich isotherm equations. The Langmuir model was more accurate for the equilibrium data of MB adsorption at pH 5.1. The PFOM and PSOM were adjusted to experimental adsorption kinetics data, being PSOM, which describes better the MB adsorption by PMS powder. This was confirmed by calculating the maximum adsorption capacity with PSOM, which was 42.7 mg g^−1^, being nearly similar of the experimental value of 43.5 mg g^−1^. The analysis of adsorption thermodynamics showed that the MB was adsorbed exothermically with a ΔH_0_ = − 20.78 kJ mol^−1^, and spontaneously with ΔG_0_ from − 0.99 to − 6.38 kJ mol^−1^ in the range of temperature from 291 to 363 K, respectively. These results confirm that the sludge from paper industry can be used as biosorbent with remarkable adsorption capacity and low cost for the treatment of wastewater. PMS can be applied in the future for the depollution of the effluents from the textile industry, which are highly charged with dyes.

## Introduction

Industries generate millions of tons of wastes each year. Their management can be difficult and expensive, and new solutions other than disposal must be developed to valorize them and reintroduce new added value products into the production chain. This option contributes with the decrease of waste emissions and the protection of human and environmental health.

Paper manufacturing generates a large quantity of wastes, and landfill is the most common option because it is simple and low expensive^1^. The global scale production in pulp and paper sector is 184.4 Mt/year of pulp and 402.790 Mt/year of paper^2,3^, which generates about 400 Mt/year of paper mill sludge^4^.

Several studies have been performed to valorize paper mill sludge (PMS), for example in the sectors of agricultural and forestry spreading^5^, composting, incineration, gasification, cement production and concrete preparation^6^, manufacture of animal feed^7^, water decontamination^8,9^, and transformation into biofuels (biogas^10^, biodiesel^11^, and bioethanol) and chemicals (isoprene)^12^.

In this context, the PMS from the paper industry has been used as an adsorbent to remove pollutants from wastewater. This residue is emitted in huge volumes and in several countries; and in some of them it is uncontrolled discharged to the environment^13,14^. When PMS is disposed in landfills, this represents particularly challenges and poses severe environmental hazards because of the high content of toxic, organic and heavy metal pollutants due to the treatment of paper at the industry like the use of dichromates for wood treatment^15^.

Dyes are important raw materials used in various industrial sectors, such as textile, paper, rubber, plastic, leather, cosmetic, pharmaceutical and food. They are mainly used to improve the appearance of final products. Annually, more than 100,000 different dyes are produced worldwide, and 8–12% of unused dyes are discharged into water bodies^16,17^. This represents a huge risk for the environment and human health because they are non-biodegradable and present properties of toxicity, carcinogenic, teratogenic and mutagenic agents^18–20^. Among these problematic dyes can be cited the textile colors^21^, reactive black 5^22^, orange G and yellow 23^23^. To control emissions of these dyes there are physical, chemical, physicochemical, and biological methods, such as membrane filtration^22,24^, advanced oxidation^25^, flocculation^21^, and fungi^26^. Among the available methods, the adsorption is a good wastewater treatment technology for removing dyes because it offers ease of use and high removal rates, combined with economic costs^27^.

Biosorption is an environmental depollution technology based on the use of high adsorption capacity of biosourced materials. The main characteristics of biosorbents is that they are derived from biomasses (microorganisms, plants and animals) and their operating principle is the capture of pollutants by the interaction of functional groups present over their surface with pollutants dissolved in water^28–32^. The presence of polar or ionizable functional groups confers the biosorbents with high affinity for charged compounds, such as metallic cations and ionic dyes. The biosorbents present several advantages in comparison to traditional adsorbents, such as they are produced by simple and low-cost processes, they have high efficiency, and they are ease to use and environmentally friendly. Whereas the traditional synthetic adsorbents are mainly manufactured from non-biosourced materials, such as synthetic polymers^33–35^, ion exchange resins^36,37^ and activated carbon^38–40^; this means that they are not biodegradable. Their production consumes great amounts of raw materials and energy and can generate toxic by-products and have net negative environmental fingerprint. The biosorbents can be classed as natural, modified, or engineered materials. Natural biosorbents are those materials that can be used in their natural form without modifying the chemical composition of their surface. To produce them it is necessary mechanical and physical conditioning units, such as grinding, sieving and drying. The biosorbents remove pollutants by the interactions created by their functional groups naturally presented on their surface with the compounds present in water. For example, the structural diversity of synthetic dyes is due to different groups of chromophores such as azo, anthraquinone and triphenylmethane^29^. These groups are responsible of interactions with biosorbents, such as the azo group (–N=N–) which creates electrostatic interactions with hydroxyl groups. These functional groups are constituents of the skeleton of biomolecules like the cellulose, which is present in the fibers of agricultural wastes, like orange peel and peanut hull^41^. Another example of interactions of biosorbents and pollutants are the adsorption of anthraquinone dye molecules on the surface of microorganism through forces such as Van der Waals, hydrogen bonding, and electrostatic interactions. These interactions are created by functional groups of microorganisms surface like hydroxyl (–OH) and amine (–NH_2_), with the hydroxyl (–OH), amino (–NH_2_) and carbonyl (–C=O) groups of anthraquinone dyes. Other examples of the use of non-modified biosorbents to remove pollutants are the non-activated biochar for removing malachite green^32^, the biomass of *Bacillus gordonae* for tectilon blue^38^, the biomass of diatom algae for metal ions and nutrients (nitrogen-N and phosphorus-P)^42^, the green algae for organic compounds and heavy metals (like uranium)^43^, the pomegranate peel for radionuclides^44^, the date palm fibers for nutrients (N and P)^45^, the microorganisms for emerging pollutants (pharmaceutical and personal care products, plasticizers, surfactants, and persistent organic pollutants)^31^, and marine algae for volatile organic compounds^46^.

The modification of biosorbent surface has the finality to increase the adsorption capacity by grafting functional groups that are not naturally present in biosorbents, or which are in low concentration. This increases the selectivity to capture specific pollutants. For example, the thermochemical functionalization of wood biochar using a mixture of inorganic acids let to increase the total acidity of non-modified biochar of 2.7 mmol g^−1^ (given by the presence of carboxylic, lactonic and phenolic acids) up to 32.7 mmol g^−1^. This changed the negative charge of biochar from − 31.4 mV for non-modified to − 53.7 mV for modified and let to increase the capture of viruses present in an aqueous solution from 69.0 to 99.7%, respectively^47^. The inconvenient of modified biosorbents is the additional consumption of chemical reagents and energy, decreasing the green aspect of modified biosorbents. Thus, an economic-benefit analysis is necessary to determine if the modification is advantageous. The engineered biosorbents are mainly biocomposites based on joining the advantages of two different materials to produce the adsorbent. In general, the biocomposite contains an inert or inorganic material that plays the role of support for the biosorbent, which offers the active surface. The aim of produce these bioengineered materials is to obtain more resistant biosorbents to mechanical forces exerted by the weight of the packed bed, shear forces of water, and thermic expansions and contractions of system. They can also confer protection to the active layer against the chemical action of corrosive and poisonous compounds. Another characteristic of engineered biosorbents could be the magnetic character given to particles to recover them easily by means of a magnetic field, for regenerating and reusing them. The use of modified and engineered biosorbents is wide, for example, the removal of ionic dyes such as malachite green with magnetized particles of biobased activated carbon, aniline blue with sodium tetraborate-modified kaolinite clay adsorbent^48^, congo red and tartrazine with cellulose modified with cetyltrimethylammonium chloride^49^, reactive black 5 and congo red with coffee waste modified with polyethylenimine^50^ and rhodamine B with diatomite modified with ethylene diamine-trimesoyl chloride^51^.

In general, biosorbents let to obtain high adsorption capacities, being competitive against traditional adsorbents, even if their specific surface is smaller. For example, the adsorption capacity of corncob for MB was reported up to 417 mg g^−1^^52^, whereas for activated carbon it was around 269 mg g^−1^^40^.

The main goal of this study was the development of new biosorbents having the advantages of low cost, widely available and with high adsorption potential for a large spectrum of pollutants. The valorization of an organic residue as biosorbent by means of some non-expensive conditioning steps let to produce new materials in a sustainable development concept. This kind of valorization produces several socioeconomic and environmental advantages, for example the decrease of wastes to landfilling, of greenhouse emissions by the biodegradation of organic residues, and capital investments to preserve the environnement. In order to reach this goal, we had the following specific objectives: (1) to show the valorization potential of residual PMS which constantly increases, and (2) to control emissions of MB as representative molecule of cationic dye high toxic pollutant, the residual MB, which is present in the effluents of textile industries**.** The selection of this pollutant is based on the capacity of MB to liberate aromatic amines such benzidine methylene, which is carcinogenic^53,54^, its presence in aquatic ecosystems can decrease the photosynthetic activity of plants because it obstructs the sunlight penetration^55^.

## Materials and methods

### Adsorbent preparation

The PMS was from the FADERCO industrial unit at Setif, which is the leader of personal hygiene products in Algeria. The PMS was dried at 105 °C (Memmert,Germany) during 24 h, and then crushed using a grinder. The powder was sieved to obtain fine particles smaller than 100 μm. The PMS powder was stored in a plastic bottle in a desiccator until further use.

### Physico-chemical characterization

The PMS was characterized determining the pH zero point charge (pHZPC), identification and quantification of elements (X-Ray fluoresence spectrometry, XRF), the functional groups on the surface (Infrared spectroscopy, FTIR) and the crystallography (X-ray Diffraction, DRX). This characterization gives us information on both structure and composition of the paper sludge, in order to determine its potential as an adsorbent of organic pollutants in water pollution control.

### Solids

To determine the profile of solids in PMS, 3 g of PMS was dried at 105 °C as indicated before to determine water and total solids content. Then dried solids were calcined at 450 °C in a muffle oven (SHIMADZU) for 2 h to determine the content of nonvolatile solids (ashes) and volatile solids (organic matter).

### pH zero point charge (pHZPC)

The pHZPC of an adsorbent is the pH at which the adsorbent surface becomes electrically neutral^56^. It was determined as follows: in ten Erlenmeyer flasks of 50 mL were put 50 mg of adsorbent. 50 ml of distilled water were added and solutions of HCl (37%, Sigma-Aldrich, Germany) or NaOH (98%, Sigma-Aldrich, Germany) were used to adjust the pH of PMS suspension from 2 to 12. The suspensions were agitated for 24 h at 200 rpm.

As soon as the stirring is over, the final pH values are taken, using a pH metre sension™ (pH 31). The ΔpH (pHf-pHi) as a function of pHi is plotted and the intersection of the ΔpH curve with the x-axis at its zero value corresponds to the pHZPC, which is the isoelectric point of PMS powder.

### Identification and quantification of elements

The qualitative and quantitative determination of elements was performed by XRF (ZSX Primus IV spectrophotometer). Seven g of PMS was crushed and pressed to form a lozenge before proceeding with the analysis. The sample is irradiated with a beam of X-rays, each element of the sample emits its own fluorescence radiation.

### Surface area of material

The specific surface area calculated by the Brunauer, Emmett and Teller theory (S_BET_) and the pore volume of dried PMS powder were determined by nitrogen gas adsorption at 77.3 K and an equilibration interval of 15 s (Micromeritics ASAP 2000, Germany).

### Morphology of PMS powder

The scanning electron microscopy (SEM) technique was employed to observe morphology of PMS dried powder. The SEM micrograph (Quanta 250FEG) was used to observe PMS morphology at different microscopic scales by electron beam bombardment. The PMS dried powder is fixed on a support for insertion into the scanning electron microscope. The electron beam is scanned horizontally and vertically over the sample surface. When the electrons in the beam interact with the sample, different signals are emitted. To explore the structure of materials at microscopic levels, a zoom of 50 and 100 um was achieved.

### Functional groups present on the surface of PMS

The determination of functional groups was performed by Fourier transform infrared analysis (FTIR) (SHIMADZU FTIR-8400 S spectrometer) before and after adsorption. The spectra were measured between 4000 and 400 cm^−1^.

### Crystalline phases

The crystalline phases of PMS were identified using X-ray diffraction (XRD) (Bruker D2). The material was bombarded with a monochromatic and parallel X-ray beam with a wavelength of 1.54060 A°, produced by a copper anticathode.

The crystal size of the material is calculated using the Scherrer equation^57^:

1 $${\text{D }} = { }\frac{K \lambda }{{\beta \cos \theta }}$$

where D: Crystallites size (nm), K: 0.9 (Scherrer constant), $\lambda$: 0.15406 (nm), wavelength of the X-ray sources, $\beta :$ FWHM (radians), $\theta :$ Peak position (radians).

The peak position and FWHM from XRD data are determined and introduced in Scherrer equation to calculate the crystal size of the PMS powder”.

### Preparation of adsorbate (the pollutant)

**Figure 1:**
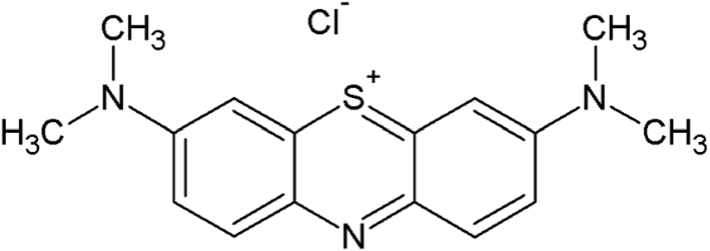
Chemical structure of MB.

The MB (Fig. 1) is a cationic dye was used as adsorbate.

A solution of 1 g L^−1^ of MB (LabKem, 89% of purity, index CI 52015, molar mass of 319.85 mol g^−1^) was prepared. The concentration of MB before and after the adsorption tests was determined using UV–visible spectrometer (Shimadzu UV–VIS, Germany) at wavelength of 664 nm that corresponds to the maximum absorbance of the MB.

### Adsorption tests

The adsorption is influenced by a variety of physicochemical parameters. Their effect on adsorption was analyzed according to the experimental plan shown on Table 1.

**Table 1:** Physico-chemical parameters.

Effect of	Parameter levels				
	Ci (ppm)	Tc (min)	pH	m (g)	T (°C)
Initial dye concentration, Ci (ppm)	90–400	1440	6.5	0.1	30
Contact time, Tc (min)	250	1–60	6.5	0.1	30
Initial pH	250	1440	2–9	0.1	30
Adsorbent mass, m (g)	250	1440	6.5	0.05–0.9	30
Temperature, T (°C)	250	1440	6.5	0.1	18–90

The adsorption assays were performed varying the initial dye concentration from 90 to 400 ppm, contact time from 1 to 60 min, initial pH from 2 to 9, mass of PMS dried powder from 0.05 to 0.9 g, and temperature from 18 to 90 °C. All the assays were performed under stirring at 200 rpm. 100 mg of adsorbent was added in 25 ml of a solution of MB with fixed initial concentration and initial pH. At the end of each assay, the suspension was filtered with cellulose filters 0.45 µm and the filtrate was analyzed using UV–visible spectrometer to determine final MB concentration.

The amount of adsorbed MB was calculated using the following equation

2 $${\mathrm{q}}_{\mathrm{t }}=\frac{{(C}_{0}-{C}_{t})}{m}*V$$

where C_0_: represents the initial dye concentration (mg L^−1^). C_t_: represents the dye concentration at any time (mg L^−1^). V: represents the volume of dye solution (L). m: represents the mass of adsorbent (g).

### Isotherm models

The adsorption models of Langmuir^58^ and Freundlich^59^ were fitted to experimental results**.** According to the Langmuir model, the equilibrium is reached when a monolayer of adsorbate molecules saturates the adsorbent. The Langmuir model is expressed as follows:

3 $$\mathrm{qe}=\frac{(\mathrm{Qm}*\mathrm{Kl}*\mathrm{Ce})}{(1+\mathrm{Kl}*\mathrm{Ce})}$$

Linearizing Eq. (3):

4 $$\frac{1}{\mathrm{qe}}=\frac{1}{\mathrm{qm}*\mathrm{Kl}}*\frac{1}{\mathrm{Ce}}+\frac{1}{\mathrm{qm}}$$

5 $$\mathrm{So}:\frac{\mathrm{Ce}}{\mathrm{qe}}=\frac{1}{\mathrm{qm}*\mathrm{Kl}}+\frac{1}{\mathrm{qm}}*\mathrm{Ce}$$

This equation allows to calculate the value of qm and K_l_ either by plotting^60^:

where C_e_: is the concentration at equilibrium (mg L^−1^). q_e_: is the quantity of product adsorbed per unit mass of adsorbent (mg g^−1^). q_m_: is the maximum theoretical adsorption capacity (mg g^−1^). k_l_: is the constant of thermodynamic absorption equilibrium (L mg^−1^).

The Freundlich’s model is based on adsorption on heterogeneous surfaces and its mathematical expression is as follows^61^:

6 $${\text{q}}_{{\text{e}}} = {\text{K}}_{{\text{f}}} {\text{Ce}}^{{{\text{1/n}}}}$$

where K_f_: is a constant that indicates the adsorption capacity of the adsorbent. n: is a constant that represents the intensity of adsorption and indicates the kind of adsorption that occurs in the system. If n < 1 the chemical adsorption is present and if n > 1 the physical adsorption is present.

The parameters K_f_ and n are determined by linearizing Eq. (5) using logarithms and plotting the resulting linear equation where log K_f_ is the y-intercept for a linear equation and 1/n is the slope.

7 $${\text{Log q}}_{{\text{e}}} = {\text{ log K}}_{{\text{f}}} + {1}/{\text{n log Ce}}$$

### Modeling of adsorption kinetics

The order of the reaction is an essential feature to define and understand the reaction process. In the present study, two kinetics models were used, the pseudo-first-order (PFO) and pseudo-second-order (PSO) models.

a. The Lagergren equation expresses the adsorption of pseudo-first-order^62^:

8 $$\frac{{\mathrm{dq}}_{\mathrm{t}}}{\mathrm{dt}}={\mathrm{K}}_{1}({\mathrm{q}}_{\mathrm{e}}-{\mathrm{q}}_{\mathrm{t}})$$

where K_1_ is the rate constant of PFO model, and q_e_ and q_t_ as defined before.

The integration of Eq. (7) for t, in the range from 0 to t, and for q, from 0 to q_t_, the integrated equation becomes:

9 $$\mathrm{log}\;\left({q}_{e}-{q}_{t}\right)=\mathrm{log}\;({q}_{e})-\frac{{k}_{1}}{2.303} \mathrm{t}$$

So:

10 $${\mathrm{ e}}^{\mathrm{log }\;({\mathrm{q}}_{\mathrm{e}}-{\mathrm{q}}_{\mathrm{t}})}={\mathrm{e}}^{\mathrm{log }\;(\mathrm{qe})}+{\mathrm{e}}^{(\frac{-{\mathrm{K}}_{{1}_{\mathrm{t}}}}{2.303})}$$

The equation becomes:

11 $${\mathrm{q}}_{\mathrm{t}}={\mathrm{q}}_{\mathrm{e}}+{\mathrm{e}}^{(\frac{-{\mathrm{K}}_{{1}_{\mathrm{t}}}}{2.303})}$$

The log plot (q_e_-q_t_) in function of (t) gives a line with a slope of—K_1_/2.303 and the y-intercept equal to log (q_e_).

b. The adsorption of pseudo-second-order is expressed by the equation^63^:

12 $$\frac{{{\text{dq}}_{{\text{t}}} }}{{{\text{dt}}}} = {\text{k }}_{2} \left( {{\text{q}}_{{\text{e}}} - {\text{q}}_{{\text{t}}} } \right)^{2}$$

where K_2_ is the rate constant of PSO model, and qe and qt as defined before.

The integration of Eq. (9) for t, in the range from 0 to t, and for q, from 0 to qt, the integrated equation becomes:

13 $$\frac{\mathrm{t}}{{\mathrm{q}}_{\mathrm{t}}}=\frac{1}{{\mathrm{k}}_{2}{{\mathrm{q}}_{\mathrm{e}}}^{2}}+\frac{1}{{\mathrm{q}}_{\mathrm{e}}}{\text{t}}$$

The equation becomes:

14 $${\mathrm{q}}_{\mathrm{t}}=\frac{\mathrm{t}}{{\mathrm{K}}_{2}{\mathrm{q}}_{\mathrm{e}}^{2}\mathrm{ t}}+{\mathrm{q}}_{\mathrm{e}}$$

The constants can be determined by plotting t/q_t_ as a function of t, which gives a line with 1/K_2_q_e_^2^ as y-intercept and 1/q_e_ as slope.

### Thermodynamic parameters

The thermodynamics of adsorption is expressed by the following equation^64^:

15 $$\mathrm{Log}\frac{{\mathrm{q}}_{\mathrm{e}}}{{\mathrm{c}}_{\mathrm{e}}}=\frac{\Delta \mathrm{S}}{2.303\mathrm{R}}+\frac{(-\Delta \mathrm{H})}{2.303\mathrm{RT}}$$

where ∆H°: is the enthalpy (kJ mol^−1^). ∆S°: is the entropy in (J mol^−1^ K^−1^). T: is the temperature (K). R: is the ideal gas constant (R = 8.314 J mol^−1^ K^−1^). q_e_: is the adsorption capacity at equilibrium (mg g^−1^). C_e_: is the equilibrium concentration (mg L^−1^).

This equation let to know the ΔH and ΔS of adsorption and the ΔG can be calculated using the general equation of thermodynamics^64^:

16 $$\Delta {\text{G}}^{0} = \, \Delta {\text{H}}^{0} {-}{\text{ T}}\Delta {\text{S}}^{0}$$

## Results and discussion

### Physicochemical characterization

#### Solids

The content of nonvolatile solids (ashes) for dried PMS powder was determined by gravimetry, being around 1.2% (w/w) and the volatile solids (VS) content was 98.8%. The VS content of PMS powder is in the range of some VS observed in literature shown in Table ST1 (all tables ST are in supplementary information file 1).

### pH zero-point charge (pHZPC)

**Figure 2:**
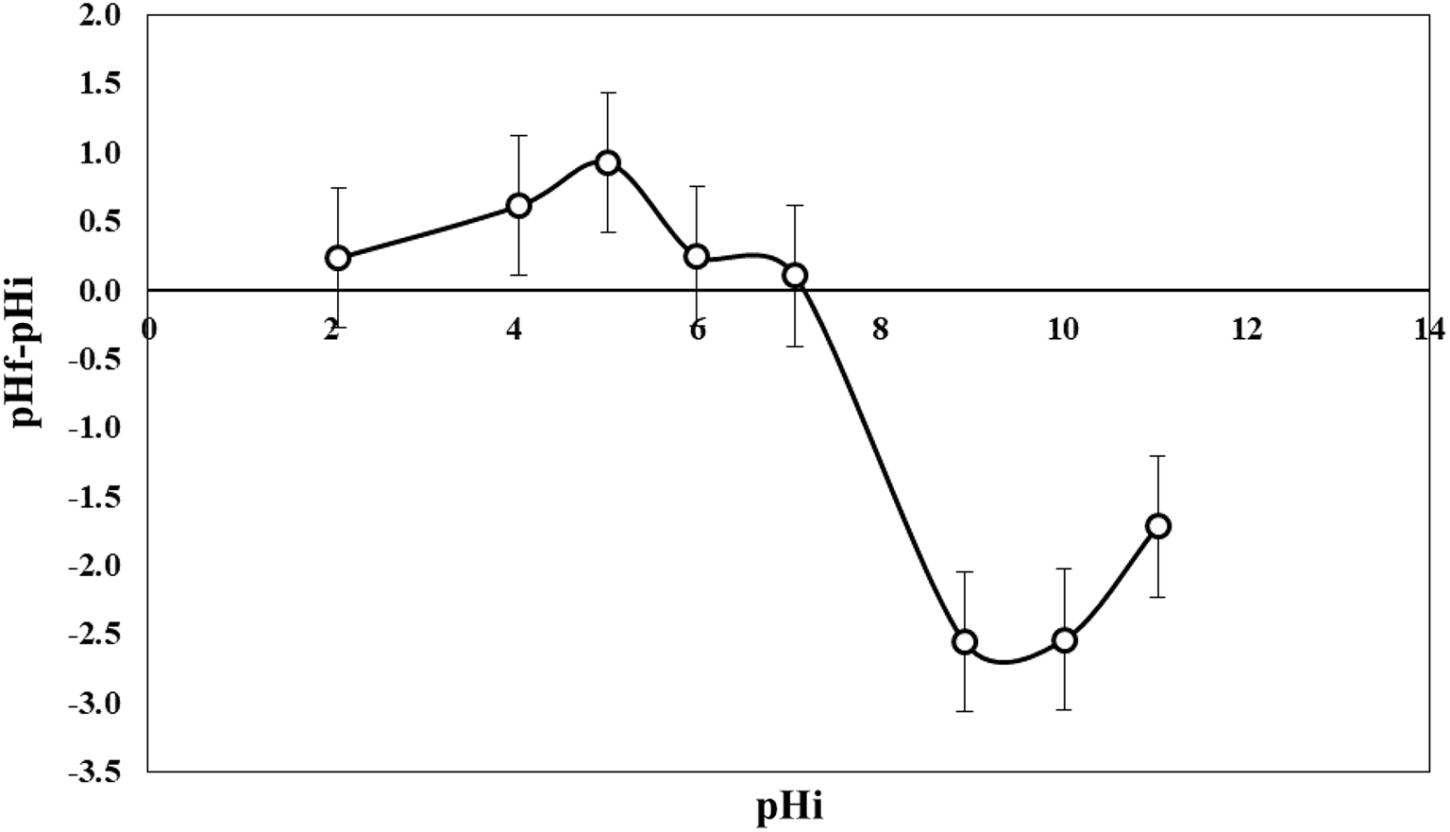
Surface charge of adsorbent as a function of pH.

Figure 2 shows the graph of ΔpH as a function of pHi. The point of zero charge for the dried PMS powder was observed at pHi of 7, 14. This point corresponds to ΔpH = 0, which means that pHf = pHi. Where the surface of the PMS powder is electrically neutral.

The range [0–7] is characterized by ΔpH > 0 (pHf > pHi). In this range, the pH of the solution was smaller than the pHZPC. The medium is acidic causing the protonation of the solution, which positively charges the surface of the PMS powder and gives it an anionic exchange capacity.

The range [7–12] is characterized by ΔpH ˂ 0 (pHf <pHi). In this range, the pH of the solution was bigger than the pHZPC. The medium is basic causing the deprotonation of the solution, which negatively charges the surface of the PMS powder and gives it a cationic exchange capacity.

### Specific surface area of PMS powder

The S_BET_ and the pore volume of a solid material are main factors that influence the adsorption capacity. The PMS had low values of these parameters; the S_BET_ was 4.3 m^2^ g^−1^ and the pore volume 0.004 cm^3^ g^−1^. For example, the S_BET_ reported for date pits was 8.1 m^2^ g^−1^^70^, and for barbecue charcoal 15.5 m^2^ g^−1^, whereas the pore volume for the latter was 0.022 cm^3^ g^−1^^70^.

### Morphology of PMS powder

**Figure 3:**
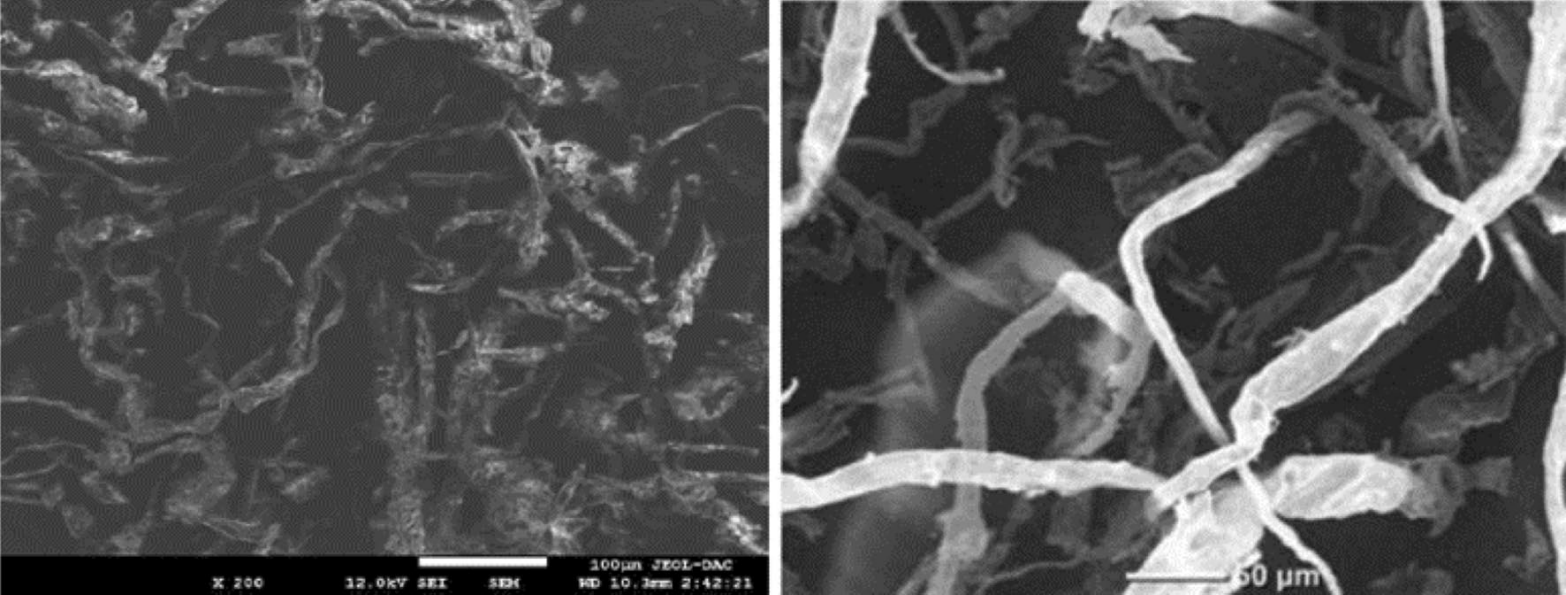
Surface morphology of PMS powder.

According to the SEM micrograph of PMS (Fig. 3), the particles presented a morphology of long and non-oriented micro-fibers^71^ with irregular structure. This increases the surface turbulence and random contact between biosorbent and MB molecules, counterbalancing the small specific surface area discussed below. This structure also increases the exposure of functional groups, which are the responsible of MB removal.

### Identification and quantification of elements

Table ST2 (supplementary information file 1) shows the elements identified in the dried PMS powder. The results show that the dried PMS powder is rich in oxygen 52.4% (w/w) and carbon 43.8% (w/w). The content of other elements, which were identified, was lower than 1% (w/w). In the case of Mn, Ni, Cu, Br, Zr and Pb, their content was lower than 0.01% (w/w), for this reason, they are not included in the Table ST2. The content of carbon and oxygen in the PMS powder of some mills reported in literature is shown in Table ST3. According to these data, the PMS used in the present study had an averaged content of these elements, being representative powder of PMS.

### Functional groups present on the surface of PMS powder

**Figure 4:**
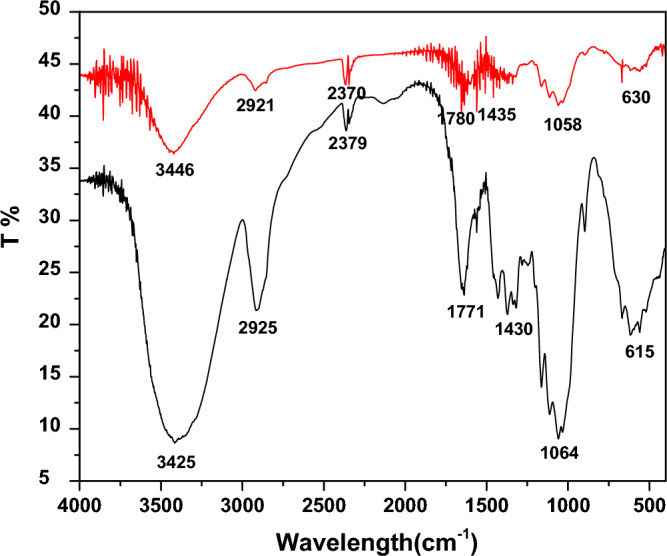
IR spectra of PMS powder: Before adsorption of MB (red line). After adsorption of MB (black line).

The Infra-Red (IR) spectra for dried PMS powder is shown in Fig. 4 before the adsorption and after the adsorption of MB. The main elongations observed in Fig. 4 are identified in Table ST4. For the PMS before the adsorption, the IR spectra presents a wide and moderate elongation band around 3446 cm^−1^ which corresponds to vibration of OH functional group, a fine and weak elongation band around 2921 cm^−1^ for the vibration of CH_methyl_ alkane functional group, a fine and moderate elongation band around 1780 cm^-1^ for the vibration of C=O functional group, a fine and weak elongation band around 1435 cm^−1^ for the vibration of C–H alkane functional group, and a fine and weak elongation band around 1058 cm^−1^ for the vibration of C–O–C functional group.

For the PMS after the adsorption, the IR spectra presents a wide and intense elongation band around 3425 cm^−1^ which corresponds to vibration of OH functional group, a fine and moderate elongation band around 2925 cm^−1^ for the vibration of CH_methyl_ alkane functional group, a fine and moderate elongation band around 1771 cm^−1^ for the vibration of C=O functional group, fine and moderate elongation band around 1430 cm^−1^ for the vibration of C–H alkane functional group, and a fine and intense elongation band around 1064 cm^−1^ for vibration of C–O–C functional group.

These changes observed between spectra in Fig. 4 (intensity of some bands) indicated the possible involvement of those functional groups on the surface of the dried paper mill sludge in sorption process.

### Crystalline phases

**Figure 5:**
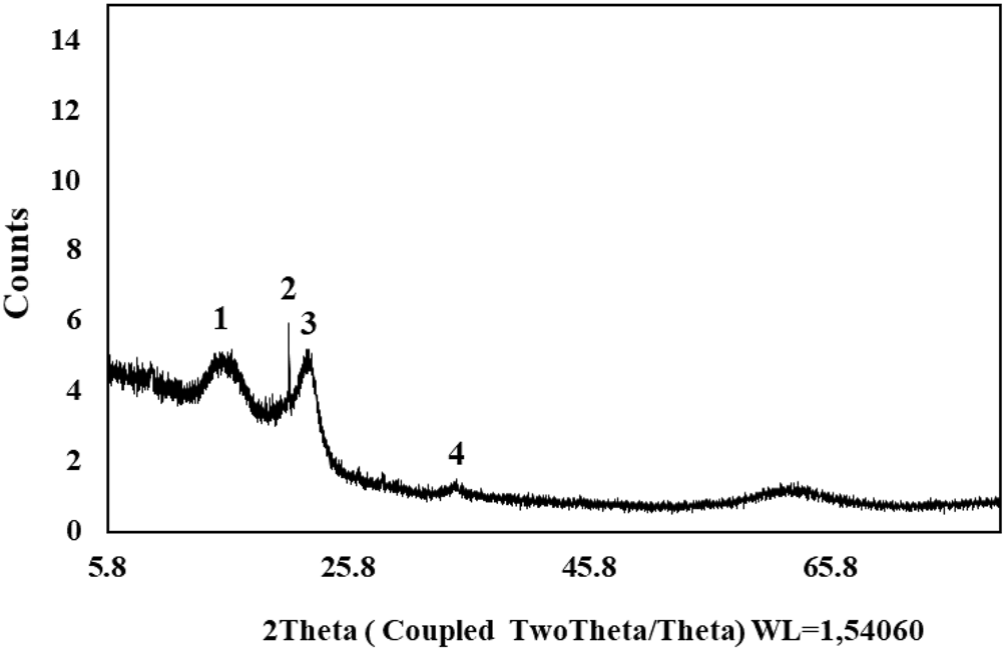
XRD spectra of the PMS powder.

Figure 5 shows the X-ray diffraction analysis of dried PMS powder. The diffraction spectrum shows a series of well-defined diffraction peaks, which correspond to predominantly well-crystallized solid phases. The phases that can be identified are the kaolinite corresponding to 2Ɵ of 12.5° (peak 1), the cellulose 2Ɵ of 20.0° (peak 2), quartz 2Ɵ of 21.9° (peak 3) and calcite 2Ɵ of 32.1° (peak 4). These phases are like those found in other pulp mill sludge^73,74^. Cellulose and lignin are the originally compounds present in PMS. The other compounds (kaolinite, quartz, and calcite) are introduced into paper sludge from many sources, such from water contaminants or residues from the paper production process. In this way, PMS is a complex mixture of different materials resulting from the papermaking process (Raw materials, chemicals additives used)^73,74^.

The lignin is the main responsible of MB adsorption due to its amorphous structure and functional groups (active sites), which can interact with the MB molecules through chemical and physical bonds, for example: phenolic, hydroxyl and methoxy groups^75^. The crystal size of the crystalline structure of cellulose was calculated around 8.4 nm using the equation of Scherrer^76^.

### Adsorption of methylene blue

#### Effect of the initial dye concentration

**Figure 6:**
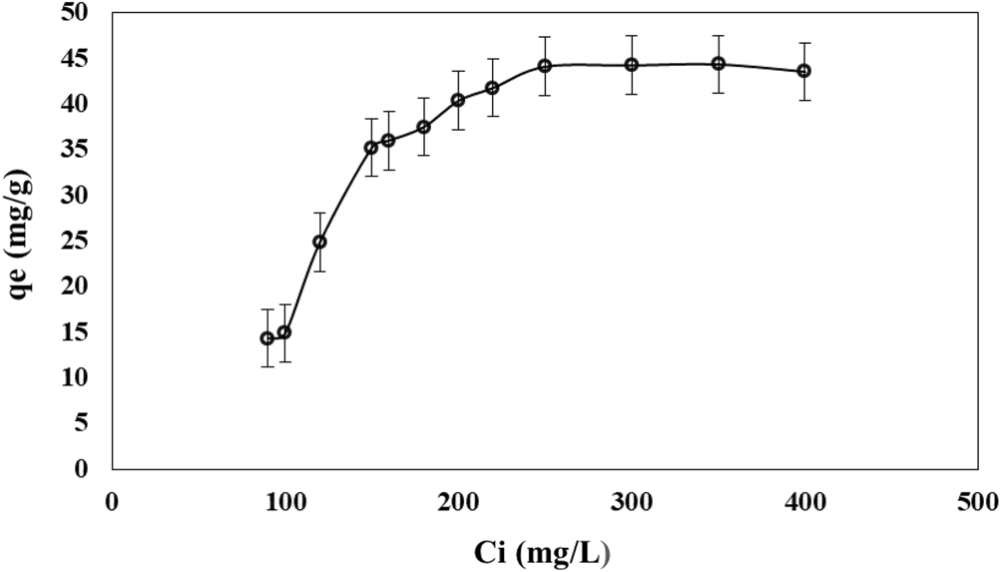
Adsorption capacity of PMS for MB as a function of the initial concentration.

Figure 6 shows the adsorption capacity of adsorbent as a function of the initial concentration of MB. The adsorption capacity increased with initial concentration of MB in the range from 90 to 250 mg L^−1^. Then, the adsorption capacity was nearly constant for initial concentration of MB higher than 250 mg L^−1^. This means that in the range from 250 to 400 mg L^−1^ the activated sites were saturated without adsorbing more molecules of MB. Thus, the maximum adsorption capacity that can be obtained with PMS powder is 45.2 mg g^−1^. Based on infrared spectra, the elongation band of C–O–C functional group increased after the adsorption of MB. This suggests that main binding site for MB is the sulfur (S) of MB skeleton shown in Fig. 1. According to the infrared spectra, a bond between the S of MB and an oxygen present in the surface of PMS is formed.

### Effect of contact time

**Figure 7:**
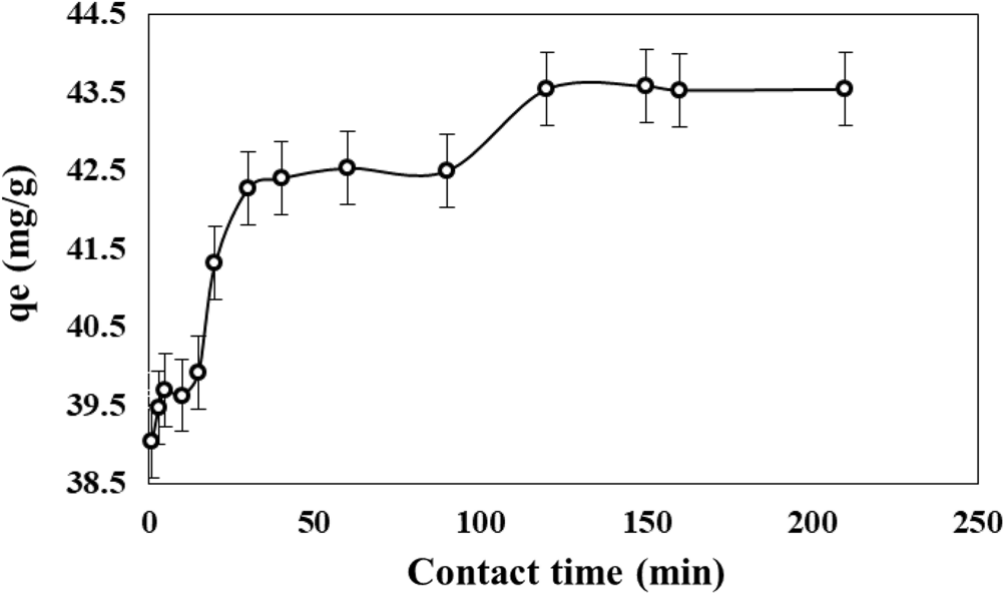
Adsorption capacity of PMS for MB as a function of the contact time.

Figure 7 shows the adsorption capacity of adsorbent as a function of the contact time. The adsorption capacity increased with the contact time, being a strong increase in the range from 1 to 30 min. Then, the adsorption capacity increased slightly after 30 min. This means that activated sites were nearly saturated at 30 min and the activated sites available will be occupied slowly until reach the equilibrium at time higher than 120 min.

According to Fig. 7, the adsorption capacity that can be obtained in short time (30 min) is 42.3 mg g^−1^, at 120 min it increased slightly to 43.5 mg g^−1^. This phenomenon can be explained by a quick adsorption step of MB on easily accessible sites, followed by a molecular diffusion towards less accessible adsorption sites, before reaching an adsorption equilibrium where all sites become occupied. Quick adsorption is caused by the high affinity (adsorbent-adsorbate).

### Effect of initial pH

**Figure 8:**
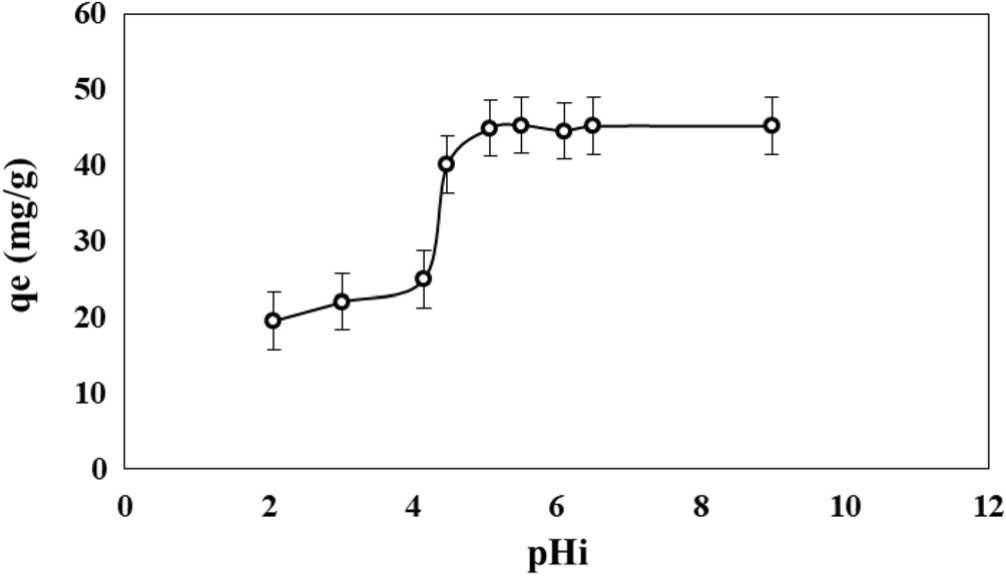
Adsorption capacity of PMS for MB as a function of the pH.

The surface properties of the adsorbent and the ionization dissociation of functional groups on the adsorbate molecule are affected by the pH of aqueous solution^77^. Figure 8 shows the adsorption capacity of adsorbent as a function of the initial pH of MB solution. The adsorption capacity increased with intial pH. Since the MB molecule is in the form of cation in the aqueous solution, it will compete with other cations for the active sites in the PMS, such as protons. The increase of pH corresponds to a decrease of protons in the solution. The concentration of 250 ppm of MB corresponds to 7.8*10^–4^ mol L^−1^, and for the same concentration of protons the solution must have a pH of 4.7. Under this pH the concentration of protons will be higher that concentration of MB and for pH above this value the concentration will be lower. As shown in Fig. 8, the MB adsorption increased slightly from pH 2.1–4.2 because the number of protons was much higher than MB molecules. The dynamic of competition for active sites will change in the range from 4.2 to 5.1, became the MB molecules preferably adsorbed at pH near of 4.5 and higher. In the range from 5.1 to 9.0 the activated sites available for MB adsorption were occupied by its molecules. Thus, the maximum adsorption capacity that can be obtained with PMS powder is 44.9 mg g^−1^, being the pH of 5.1 the lowest pH, which let to adsorb the highest amount of MB.

### Effect of adsorbent dosage on MB removal

**Figure 9:**
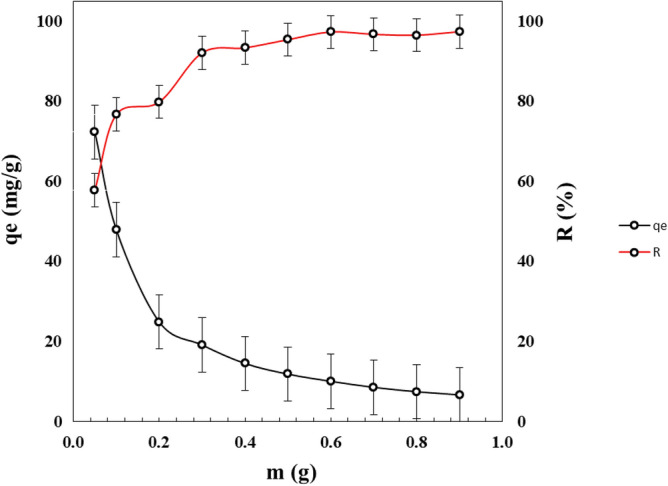
Adsorption capacity of PMS for MB and removal percentage as a function of adsorbent dosage.

Figure 9 shows the adsorption capacity of PMS and the MB removal percentage as a function of the PMS adsorbent dosage. The adsorption capacity decreased in the range from 72.3 to 6.8 mg g^−1^ with increasing adsorbent dosage from 0.05 to 0.90 g in a constant volume of 25 mL. The addition of more adsorbent in a solution containing a fixed concentration of MB will decrease the ratio of MB molecules/active sites. This will cause that the adsorption capacity will decrease even if more MB has been adsorbed. The latter was confirmed by the color removal, which increased with PMS adsorbent dosage from 58 to 98%.

The maximum adsorption capacity for PMS powder was 72.3 mg g^−1^ for a dosage of 0.05 g in 25 mL of MB solution. However, this adsorption capacity corresponds to low color removal of 58%. The best dosage of PMS in terms of MB removal, according to a practical point of view, was 0.3 g.

### Effect of temperature

**Figure 10:**
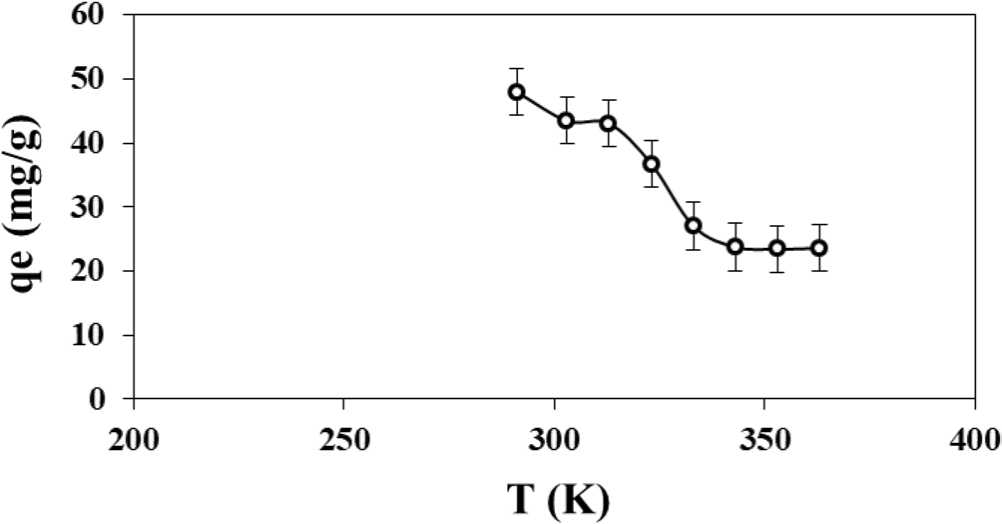
Adsorption capacity of PMS for MB as a function of the temperature.

Figure 10 shows the adsorption capacity of adsorbent as a function of temperature decreasing in the range from 291 to 343 K. Then, the adsorption capacity was nearly constant for temperature higher than 343 K. The best adsorption capacity of PMS for MB was 50 mg g^−1^ at 291 K, which was the room temperature. The adsorption occurred spontaneously without needing to heat, being an exothermic reaction. This means that the rise in temperature decreases the dye removal because the strength of the bonds between the adsorbent active sites and the MB molecules weakens^78^.

### Isotherm models

**Figure 11:**
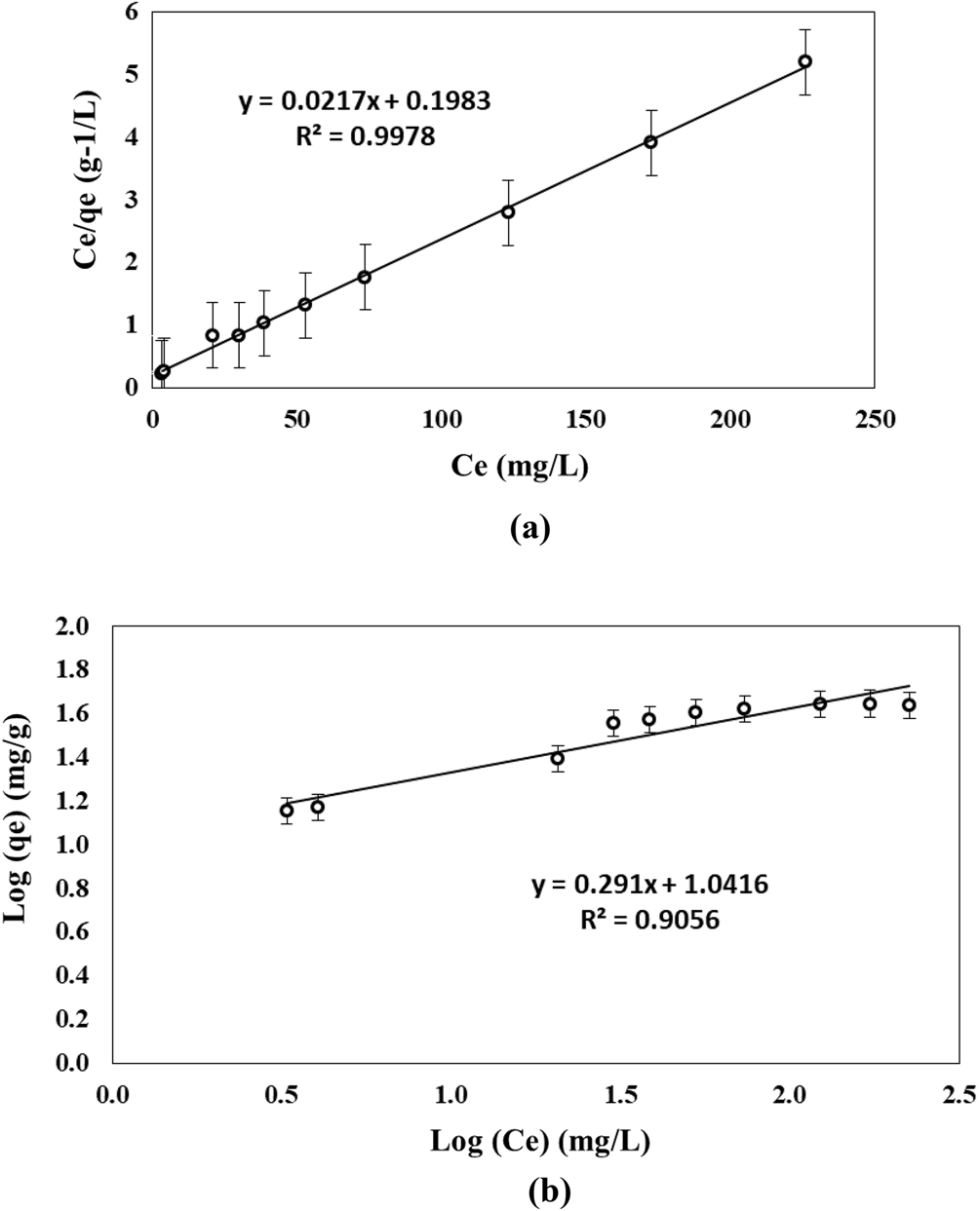
Linear representation using experimental data of (**a**) Langmuir isotherm, and (**b**) Freundlich isotherm.

Table 2 shows the results for the two models of adsorption, Langmuir and Freundlich. The Langmuir model, which describes a monolayer adsorption, was the model, which best fitted to experimental data (Fig. 11), showing a correlation factor R^2^ of 0.99. The theoretical adsorption capacity according to Langmuir was 46.1 mg g^−1^, which was close to the experimental value observed of 45.2 mg g^−1^. This suggests the formation of a monomolecular layer of adsorbate, which is due to a rapid decrease of intermolecular forces. The Langmuir model confirms that the adsorption reaction was instantaneous and reversible, the absence of interaction between the adsorbed species and the presence of a homogeneous distribution of the adsorption energies^79^.

**Table 2:** Isotherm adsorption’s parameters.

Langmuir			Freundlich			
q_max_ (mg g^−1^)	K_l_ (L mg^−1^)	R^2^	q_max_ (mg g^−1^)	K_f_	1/n	R^2^
46.1	0.109	0.99	54.7	11.005	0.29	0.91

### Modeling of adsorption kinetics

**Figure 12:**
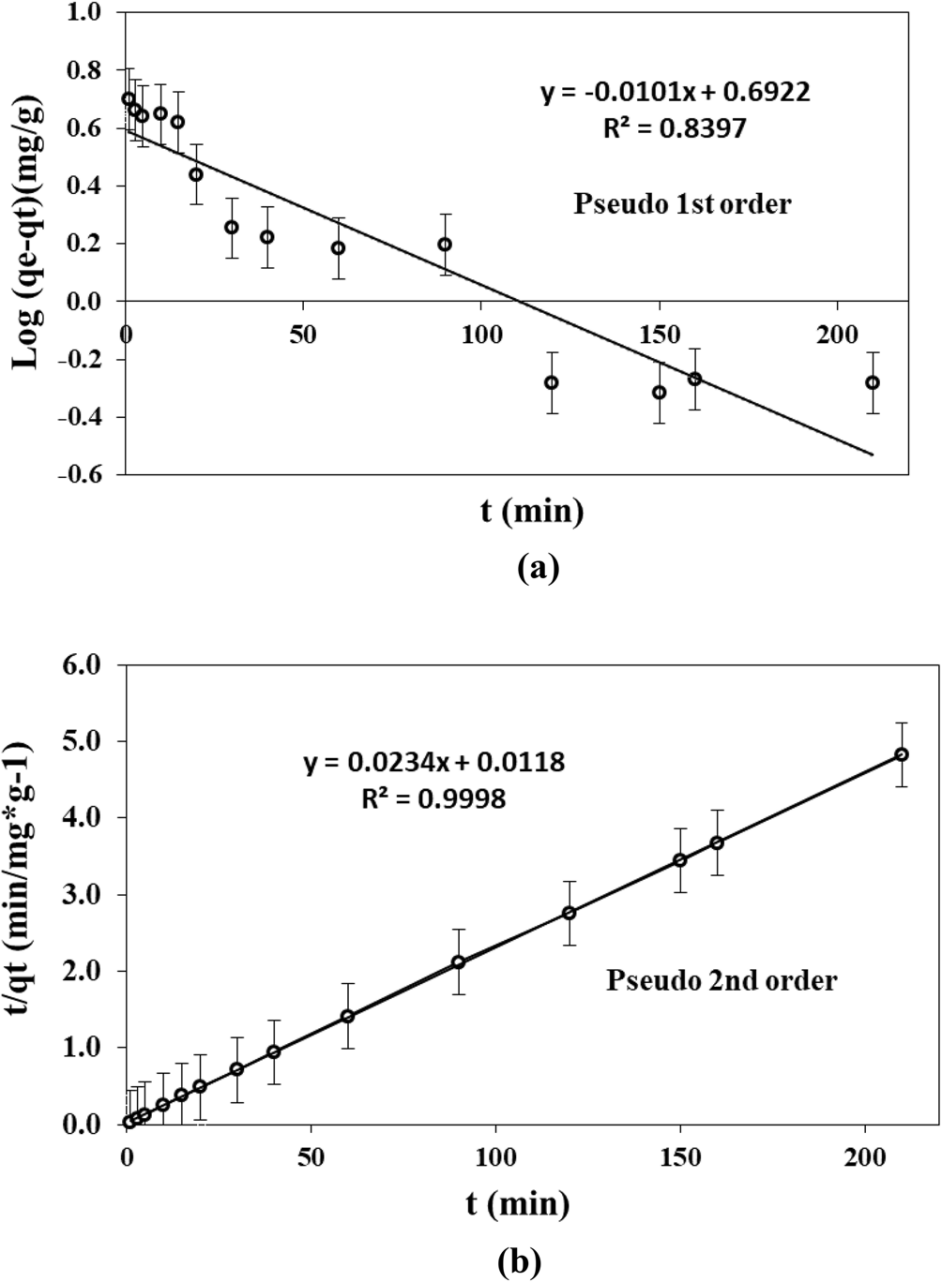
Linearized representation of (**a**) Pseudo-first order model (PFOM), and (**b**) pseudo second order model (PSOM) using experimental data.

The Pseudo first order model (PFOM) and the Pseudo second order model (PSOM) were adjusted to experimental data of kinetics adsorption (Fig. 12). The parameters for PFOM and PSOM obtained by linear regression are shown in Table 3. Figure 12 shows that the PSOM fitted better than PFOM to adsorption capacity of PMS, with a determination coefficient R^2^ of 0.99. The adsorption capacity calculated using the PSOM was 42.7 mg g^−1^, which was closer to the experimental value of 43.5 mg g^−1^ than that of PFOM, which was only 1.1 mg g^−1^.

**Table 3:** Kinetics parameters of Pseudo first order model (PFOM) and Pseudo second order model (PSOM) for MB blue adsorption.

PFOM			PSOM		
qe* (mg g^−1^)	K_1_	R^2^	qe* (mg g^−1^)	K_2_	R^2^
1.1	0.002	0.84	42.7	0.046	0.99

*q_e_ was calculated by adjusting PFOM or PSOM to experimental data.

### Thermodynamic parameters

**Figure 13:**
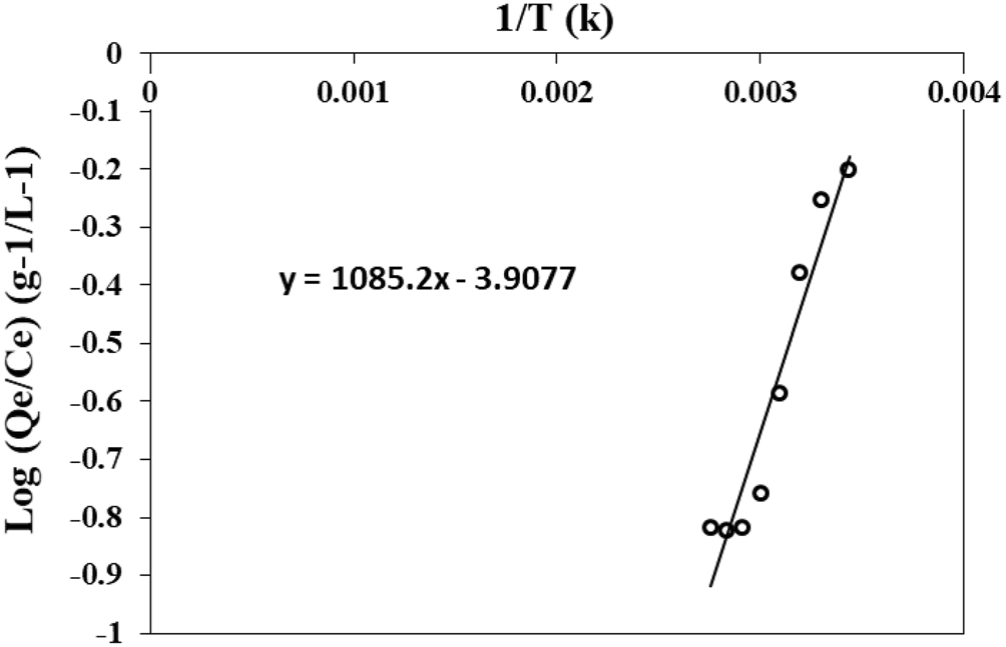
Graphical representation of linearized thermodynamic model.

Table 4 shows the thermodynamic parameters of adsorption capacity of PMS powder for MB. The ΔG calculated with Eq. (16) was always negative in the range of temperature from 291 to 363 K. This indicates that the MB adsorption process on dried PMS particles was a physisorption^80^ and a spontaneous phenomenon^81,82^. ΔH and ΔS were calculated using the y-intercept and the slope of the Eq. (15) adjusted to experimental data (Fig. 13) with a R^2^ of 0.92. The ΔH°s was − 20.778 kJ mol^−1^, and the ΔS° − 74.821 J K^−1^ mol^−1^. The negative values of ΔH and ΔS indicates that the adsorption of MB was exothermic and a random decrease of molecular interactions between adsorbate-adsorbent on the solid-solution interface^83^.

**Table 4:** Thermodynamic parameters of MB adsorption on PMS.

C = 250 mg L^−1^; m = 0.1 g; t = 30 min				
T (K)	1/T(K)	Log (qe/Ce)	ΔG° (kJ/mol)	K
291	0.0034364	− 0.2012638	− 0.994	1.205
303	0.0033003	− 0.2533605	− 1.892	2.120
313	0.0319490	− 0.3793729	− 2.640	2.758
323	0.0030910	− 0.5867813	− 3.388	2.532
333	0.0030030	− 0.7582191	− 4.136	4.456
343	0.0029154	− 0.8181247	− 4.885	5.546
353	0.0028329	− 0.8222568	− 5.633	6.817
363	0.0027548	− 0.8191124	− 6.381	8.285

### Representativeness of this study with other studies on MB adsorption by biosorbents

The biosorption of MB has been studied by other biosorbents, such as tea wastes, olive pomace, cereal husk (rice, wheat, etc.), fruit peel (orange, banana), sawdust (cherry, spruce, etc.) and seaweed. The adsorption capacity reported for these residues was observed in the range from 85.2 mg g^−1^ for tea waste to 5.2 mg g^−1^ for seaweed.

The PMS has high potential to be used as biosorbent thanks to its adsorption capacity of 72.3 mg g^−1^ under the optimal conditions determined in this study. As shown in Table 5, the adsorption capacity of PMS was slightly lower than the best of biosorbents reported in literature (tea waste) and it presents an adsorption capacity for MB in the range of activated carbon from 47.2–269.3 mg g^−1^.

**Table 5:** Adsorption capacity of biosorbents for MB.

Adsorbent material	Sorption capacity Q_0_	References
Tea waste	85.2	^84^
Olive pomace	42.3	^85^
Rice husk	40.6	^86^
Cherry sawdust	39.8	^87^
Wheat shells	16.6–21.5	^88^
Orange peel	21.1	^89^
Banana peel	20.8	^90^
Cereal chaff	20.3	^91^
*Picea abies*	17.9	^92^
Seaweed	5.2	^93^
Paper mill sludge	72.3	This study
Activated carbon	47.2–269.3	^39,40^

## Conclusions

The adsorption of MB onto PMS powder particles was studied. The PMS issued from paper industry presented high potential to be used as biosorbent to remove dyes from aqueous solutions. The optimal range of initial concentration of MB, contact time, initial pH, temperature and PMS dose on adsorption capacity was determined. The maximum adsorption capacity was 72.3 mg g^−1^, under a MB concentration of 250 mg L^−1^, 30 min, pH of 5.1, 18 °C and 0.05 mg of biosorbent in 25 mL of solution. This removal capacity is competitive with other biosorbents and even with activated carbon. According to this study, the use of PMS let to remove up to 98% of the color given by MB to wastewater. The MB adsorption on PMS powder followed a Langmuir behavior and a pseudo second order kinetics, suggesting that adsorption is a physicochemical process, and the adsorbent particles could be regenerated for being reused in new adsorption cycles. The MB adsorption is a spontaneous and exothermic process; this means that for regenerating is necessary to introduce an energy source.

The high removal capacity of PMS suggests that this material is a good option to depollute effluents charged with dyes; for example, textile sector which is the main consumer of MB. In addition, the use of a byproduct as adsorbent contributes to sustainable development of regions. This circular economy concept focuses on preserving the environment. The promising use of PMS as biosorbent opens the door to deepen the scientific research in the use of emergent materials to remove other pollutants.

### Supplementary Information

Supplementary Information 1.

Supplementary Information 2.

## Data Availability

All data generated or analysed during this study are included in this published article [and its supplementary information files] on the Supplementary file 2 as “Datasets” file.
