# Supplementary material for: High biosorption of cationic dye onto a novel material based on paper mill sludge
Source: Sci Rep. 2023 Sep 23;13:15926. doi: 10.1038/s41598-023-43032-x (PMC10518001; doi:10.1038/s41598-023-43032-x)
Supplement: Supplementary file 1 — Supplementary Information 1. [file 41598_2023_43032_MOESM1_ESM.docx]

**Table ST1** Content of volatile solids and ashes for PMS powder for some mills in the world

| **Origin** | **nonvolatile solids (ashes) % (w/w)** | **volatile solids (VS)**  **% (w/w)** | **References** |
| --- | --- | --- | --- |
| **Coimbatore (India)** | 30 | 62.5 | [65] |
| **Leon (Spain)** | 55.3 | 36.1 | [66] |
| **Hsinchu (Taiwan)** | 30 | 70 | [67] |
| **Setif (Algeria)** | 1.2 | 98.8 | **This study** |

**Table ST2** Chemical analysis by X-ray fluorescence of crude sludge

**(Elements expressed in % by mass)**

| **Element** | **Content (%, w/w)** | **Element** | **Content (%, w/w)** |
| --- | --- | --- | --- |
| **B** | 0.74 | **Cl** | 0.09 |
| **C** | 43.80 | **K** | 0.08 |
| **O** | 52.40 | **Ca** | 0.38 |
| **Mg** | 0.26 | **Ti** | 0.02 |
| **Al** | 0.52 | **Cr** | 0.06 |
| **Si** | 0.94 | **Fe** | 0.35 |
| **P** | 0.12 | **S** | 0.16 |

**Table ST3** Content of carbon and oxygen for paper mill sludge powder for some mills in the world

| **Origin** | **Carbon content % (w/w)** | **Oxygen content % (w/w)** | **References** |
| --- | --- | --- | --- |
| **Coimbatore (India)** | 29.7 | 31.6 | [65] |
| **Örnsköldsvik (Sweden)** | 44.9-48.4 | 23.6-24.4 | [68] |
| **Leon (Spain)** | 14.8 | 27.9 | [66] |
| **Athens (USA)** | 34.6 | 44.4 | [69] |
| **Setif (Algeria)** | 43.8 | 52.4 | **This study** |

**Table ST4** Functional groups of dried PMS powder before and after adsorption

| **Functional group** | **Wavenumber of dried PMS powder before the adsorption of MB (cm^-1^)** | **Wavenumber of dried PMS powder after the adsorption of MB (cm^-1^)** | **Wavenumber**  **Reported in**  **Litterature** [72] |
| --- | --- | --- | --- |
| **Hydroxyl O-H** | 3446 | 3425 | 3550-3200 |
| **Alkane_methyl_ C-H** | 2921 | 2925 | 3000-2840 |
| **Phenyl_Ester_ C=O** | 1780 | 1771 | 1770-1780 |
| **Alkane C-H** | 1435 | 1430 | 1450-1375 |
| **Vinyl_Ether_  C-O-C** | 1058 | 1064 | 1075-1020 |
