# Supplementary material for: High biosorption of cationic dye onto a novel material based on paper mill sludge
Source: Sci Rep. 2023 Sep 23;13:15926. doi: 10.1038/s41598-023-43032-x (PMC10518001; doi:10.1038/s41598-023-43032-x)
Supplement: Supplementary file 2 — Supplementary Information 2. [file 41598_2023_43032_MOESM2_ESM.docx]

**The Data of different figures:**

**The Data of: Fig. 2 Surface charge of adsorbent as a function of pH**

**Fig. 2** Surface charge of adsorbent as a function of pH

| **pHi** | **pH_f_-pH_i_** |
| --- | --- |
| 2.1  4.1  5.0  6.0  7.1  8.9  10.0  11.0 | 0.2  0.6  0.9  0.2  0.1  -2.6  -2.5  -1.7 |

**The Data of: Fig. 6 Adsorption capacity of PMS for MB as a function of the initial concentration**

**Fig. 5** Adsorption capacity of PMS for MB as a function of the initial concentration

| **Ci (mg/L)** | **qe (mg/g)** |
| --- | --- |
| 90  100  120  150  160  180  200  220  250  300  350  400 | 14.32  14.87  24.82  35.19  35.98  37.46  40.33  41.75  44.07  44.21  44.32  43.52 |

**The Data of: Fig. 7 Adsorption capacity of PMS for MB as a function of the contact time**

| **t (min)** | **qe (mg/g)** |
| --- | --- |
| 1  3  5  10  15  20  30  40  60  90  120  150  160  210 | 39.04  39.47  39.69  39.63  39.92  41.32  42.28  42.41  42.54  42.50  43.55  43.59  43.53  43.55 |

**The Data of: Fig. 8 Adsorption capacity of PMS for MB as a function of the pH**

| **pHi** | **qe (mg/g)** |
| --- | --- |
| 2.1  3.0  4.2  4.5  5.1  5.5  6.1  6.5  8.9 | 19.57  22.05  25.06  40.12  44.93  45.35  44.59  45.22  45.22 |

**The Data of: Fig. 9 Adsorption capacity of PMS for MB and removal percentage as a function of adsorbent dosage**

| **m (g)** | **qe (mg/g)** | **R (%)** |
| --- | --- | --- |
| 0.05  0.1  0.2  0.3  0.4  0.5  0.6  0.7  0.8  0.9 | 72.36  47.97  24.96  19.19  14.59  11.93  10.14  8.64  7.54  6.77 | 57.88  76.75  79.87  92.16  93.40  95.45  97.36  96.77  96.56  97.44 |

**The Data of: Fig. 10 Adsorption capacity of PMS for MB as a function of the temperature**

| **T (K)** | **qe (mg/g)** |
| --- | --- |
| 291  303  313  323  333  343  353  363 | 47.96  43.44  43.04  36.73  27.04  23.76  23.43  23.59 |

**The Data of: Fig. 11 Linear representation using experimental data of (a) Langmuir isotherm, and (b) Freundlich isotherm**

**Langmuir**

| **Ce (mg/L)** | **Ce/qe (g^-1^/L)** |
| --- | --- |
| \| 3.27 \| \| --- \| \| 4.05 \| \| 20.73 \| \| 30.16 \| \| 38.69 \| \| 52.98 \| \| 73.72 \| \| 123.14 \| \| 172.72 \| \| 225.92 \| | \| 0.23 \| \| --- \| \| 0.27 \| \| 0.83 \| \| 0.84 \| \| 1.03 \| \| 1.31 \| \| 1.76 \| \| 2.79 \| \| 3.91 \| \| 5.19 \| |

**Freundlich**

| **Log Ce (mg/L)** | **Log qe (mg/g)** |
| --- | --- |
| \| 0.51 \| \| --- \| \| 0.61 \| \| 1.32 \| \| 1.48 \| \| 1.59 \| \| 1.72 \| \| 1.87 \| \| 2.09 \| \| 2.24 \| \| 2.35 \| | \| 1.15 \| \| --- \| \| 1.17 \| \| 1.39 \| \| 1.56 \| \| 1.57 \| \| 1.60 \| \| 1.62 \| \| 1.64 \| \| 1.64 \| \| 1.64 \| |

**The Data for: Fig. 12 Linearized representation of (a) Pseudo-first order model (PFOM), and (b) pseudo second order model (PSOM) using experimental data**

| **t (min)** | **Log (qe-qt) (mg/g)** |
| --- | --- |
| \| 1 \| \| --- \| \| 3 \| \| 5 \| \| 10 \| \| 15 \| \| 20 \| \| 30 \| \| 40 \| \| 60 \| \| 90 \| \| 120 \| \| 150 \| \| 160 \| \| 210 \| | 0.70  0.66  0.64  0.65  0.62  0.44  0.25  0.22  0.19  0.19  -0.28  -0.31  -0.27  -0.28 |

**Pseudo second order model (PSOM)**

| **T (min)** | **t/qt (min/mg*g^-1^)** |
| --- | --- |
| 1  3  5  10  15  20  30  40  60  90  120  150  160  210 | 0.02  0.08  0.12  0.25  0.37  0.48  0.71  0.94  1.41  2.12  2.75  3.44  3.67  4.82 |

**The Data for: Fig. 13 Linear curve of the thermodynamic model**

| **1/T (K)** | **Log (qe/Ce) (g^-1^ /L^-1^)** |
| --- | --- |
| 0.0034  0.0033  0.0032  0.0031  0.0030  0.0029  0.0028  0.0027 | -0.201  -0.253  -0.379  -0.587  -0.758  -0.818  -0.822  -0.819 |

**Note:** The figures 3 (Fig**. 3** Surface morphology of PMS powder), 4 (**Fig. 4** IR spectra of PMS powder: Before adsorption of MB ̶̶̶̶̶̶̶ ̶̶ ̶̶̶̶̶̶̶ ̶̶ After adsorption of MB ̶̶̶̶̶̶̶ ̶̶ ̶̶̶̶̶̶̶ ̶̶), 5 (**Fig. 5** XRD spectra of the PMS powder) have been traced using equipment.
